# Author Correction: Efficient interatomic descriptors for accurate machine learning force fields of extended molecules

**DOI:** 10.1038/s41467-023-39798-3

**Published:** 2023-07-11

**Authors:** Adil Kabylda, Valentin Vassilev-Galindo, Stefan Chmiela, Igor Poltavsky, Alexandre Tkatchenko

**Affiliations:** 1 Department of Physics and Materials Science, University of Luxembourg, L-1511 Luxembourg City, Luxembourg; 2 Machine Learning Group, Technische Universität Berlin, 10587 Berlin, Germany; 3 BIFOLD – Berlin Institute for the Foundations of Learning and Data, 10587 Berlin, Germany

**Keywords:** Molecular dynamics, Computational chemistry, Quantum chemistry

Correction to: *Nature Communications* 10.1038/s41467-023-39214-w, published online 15 June 2023

The original version of this Article contained some errors in the References. In references 12, 13, 19, 49 and 52 the article numbers were incorrectly given as ‘1’. This has been replaced with the correct article numbers in the corrected version. In the original version of the Article, Reference 74 was a duplicate of Reference 70, and Reference 75 was a duplicate of Reference 71. These have been removed in the corrected version. After removing References 74 and 75, Reference 76 in the original article has been renumbered to 74. These errors have been corrected in both the HTML and PDF versions of the article.

