# Supplementary material for: Efficient interatomic descriptors for accurate machine learning force fields of extended molecules
Source: Nat Commun. 2023 Jun 15;14:3562. doi: 10.1038/s41467-023-39214-w (PMC10272221; doi:10.1038/s41467-023-39214-w)
Supplement: Supplementary file 1 — Supplementary Information [file 41467_2023_39214_MOESM1_ESM.pdf]

Supplementary Material for Efficient Interatomic Descriptors for Accurate Machine Learning Force Fields of Extended Molecules

Adil Kabylda,[†] Valentin Vassilev-Galindo,[†] Stefan Chmiela,[‡] Igor Poltavsky,[†] and Alexandre Tkatchenko[*,†]

†*Department of Physics and Materials Science, University of Luxembourg, L-1511 Luxembourg City, Luxembourg*

‡*Machine Learning Group, Technische Universität Berlin, 10587 Berlin, Germany*

E-mail: alexandre.tkatchenko@uni.lu

Supplementary Note 1.

Prediction Accuracy of Models Trained with Global, Local and Reduced descriptors. Here we compare the performance of an ML model trained using an reduced descriptor to ML models trained using global and local descriptors. Fig. 1 shows distributions of force errors for default GDML models (ML_{global}), GDML models using reduced descriptors (with 40% of the original features) as obtained from the results in Fig. 2 (ML_{opt}), and GAP/SOAP models with a cutoff of 5 Å (ML_{SOAP}) trained on 1000 training configurations for the Ac-Ala3-NHMe, the AT-AT dimer, and the buckyball catcher. Force error histograms show that the accuracy of the local ML_{SOAP} models, with respect to the ML_{global} and ML_{opt} ones, is lower with the increasing size and flexibility of the molecule. ML_{SOAP} models start with an almost equal distribution as all other models for Ac-Ala3-NHMe (Fig. 1A) but show considerably bigger errors than the ML_{global} and ML_{opt} models for the buckyball catcher (Fig. 1C). The lower accuracy of the local models is the result of neglecting non-local interactions that become prominent for the larger and more flexible systems. Regarding the ML_{opt} models, they present almost the same population of small force errors [under an absolute value of 1.0 kcal (mol Å)$^{-1}$] as the ML_{global} model, while having a lower frequency of larger errors. For instance, errors above absolute values of 3.0 and 1.0 kcal (mol Å)$^{-1}$ for the AT-AT dimer (Fig. 1B) and the buckyball catcher (Fig. 1C), respectively, are more common with the ML_{global} model. Hence, ML models constructed using reduced descriptors provide more reliable predictions than typical global and local ML models when reconstructing complex PESs.

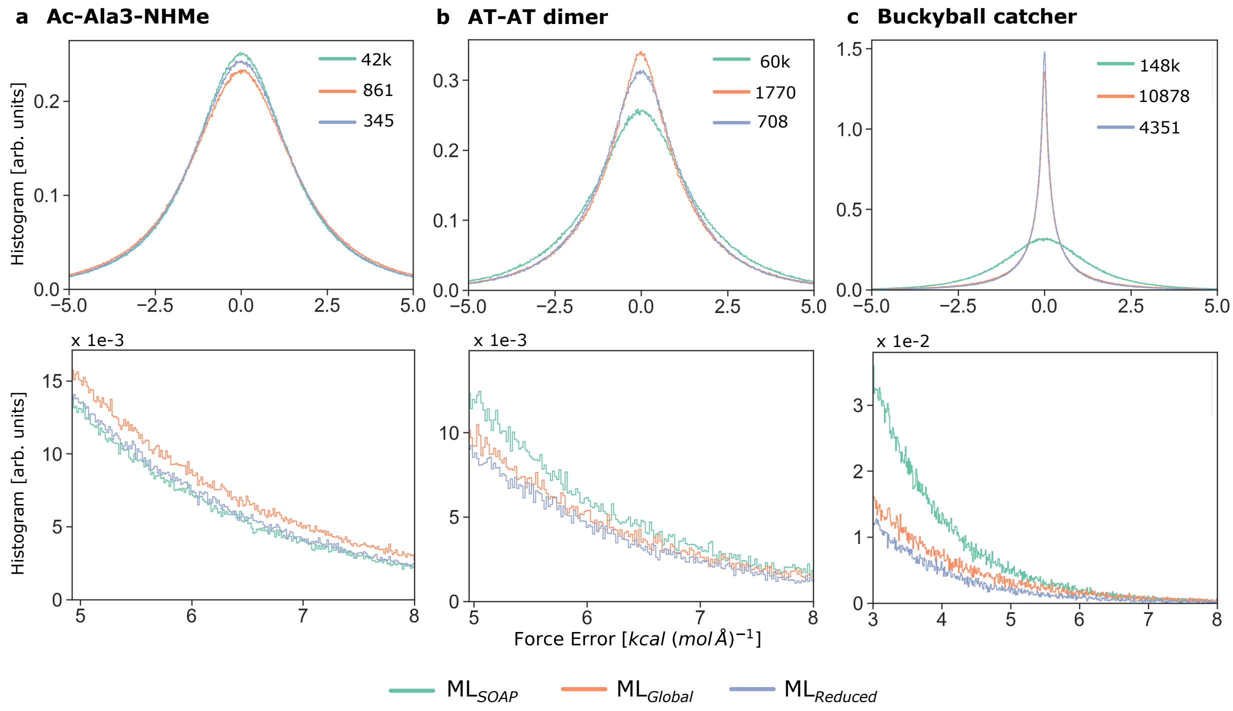

Supplementary Figure 1. Histogram of force errors [in kcal (mol Å)$^{-1}$] of the global (ML$_{Global}$), optimally reduced (ML$_{Reduced}$), and Gaussian Approximation Potential/Smooth Overlap of Atomic Positions (GAP/SOAP, ML$_{SOAP}$) models for Ac-Ala3-NHMe (**a**), the AT-AT dimer (**b**), and the buckyball catcher (**c**). The size of the descriptor of the models is given in the legend box of the figures. Upper row: section of the distribution of errors between -5 and 5 kcal (mol Å)$^{-1}$; lower row: section of the distribution in the tail from 5 to 8 (**a, b**) and from 3 to 8 (**c**) kcal (mol Å)$^{-1}$.

Supplementary Note 2.

Prediction of outlier data. We investigated the performance of global and reduced models trained on "extended" structures when tested on "compact" structures of the tetrapeptide. To perform the comparison, we have splitted the dataset based on the distance between the furthest atoms in each structure (ranges from ∼8 to ∼14 $\mathring{A}$).

We selected a threshold of 12 $\mathring{A}$ which separates clusters of compact and extended structures. With this threshold, we splitted the dataset into dataset 1 ($max(R_{ij}) < 12$ $\mathring{A}$, ∼80% of the initial dataset - 69k structures) and dataset 2 ($max(R_{ij}) \geq 12$ $\mathring{A}$, 20% - 16k structures). We used 1000 points for the training and 1000 for the validation of the models from the dataset 1 (training set - compact) and used all structures from the dataset 2 for testing (E/F RMSE extended).

To check how global and reduced models trained on "extended" structures perform on "compact" structures we repeated the same procedure with a threshold of 9.5 $\mathring{A}$, resulting in the dataset 3 ($max(R_{ij}) > 9.5$ $\mathring{A}$, ∼80% of the initial dataset) used for training (training set - extended) and dataset 4 ($max(R_{ij}) \leq 9.5$ $\mathring{A}$, 20%) used for testing (E/F RMSE compact).

Supplementary Table 1. Performance comparison of global and reduced models. Energy and Force root mean square errors (E RMSE and F RMSE) are reported in kcal mol^{-1} and kcal (mol Å)$^{-1}$, respectively. The definitions of compact and extended training sets are explained in this subsection. The Gradient Domain Machine Learning model with default global descriptor is denoted as ML_{Global}, and the model with a descriptor reduced by 60% is denoted as $ML_{R0.4}$.

Training set selected from	Model	E RMSE extended	F RMSE extended	E RMSE compact	F RMSE compact
compact	ML_{Global}	14.0	4.31	1.64	2.41
compact	$ML_{R0.4}$	7.55	3.55	1.47	2.24
extended	ML_{Global}	1.74	2.44	4.72	3.09
extended	$ML_{R0.4}$	1.53	2.29	3.05	2.67

The comparison of the Force/Energy RMSEs shows that the reduced models are more accurate than global models when dealing with "unseen" outlier structures. We can attribute such an improvement to the ability of reduced models to obtain a better description of the

environments of the molecule. This means that reduced models can better identify similar structural moities between “compact” and “extended” structures while keeping the relevant information for describing non-local interactions.

Supplementary Table 2. Relative deployment speed of the models trained on 1000 configurations. Descriptor sizes go from 1 to 0, where 1 corresponds to a default global descriptor and 0 to an empty descriptor.

Descriptor size	Ac-Ala3-NHMe	AT-AT	Buckyball Catcher
1	1.00	1.00	1.00
0.9	1.06	1.04	1.10
0.8	1.19	1.17	1.23
0.7	1.32	1.28	1.33
0.6	1.48	1.47	1.50
0.5	1.68	1.72	1.77
0.4	1.93	2.06	2.10
0.3	2.22	2.56	2.63
0.2	2.62	3.22	3.83
0.1	3.17	4.23	4.90

Supplementary Figures.

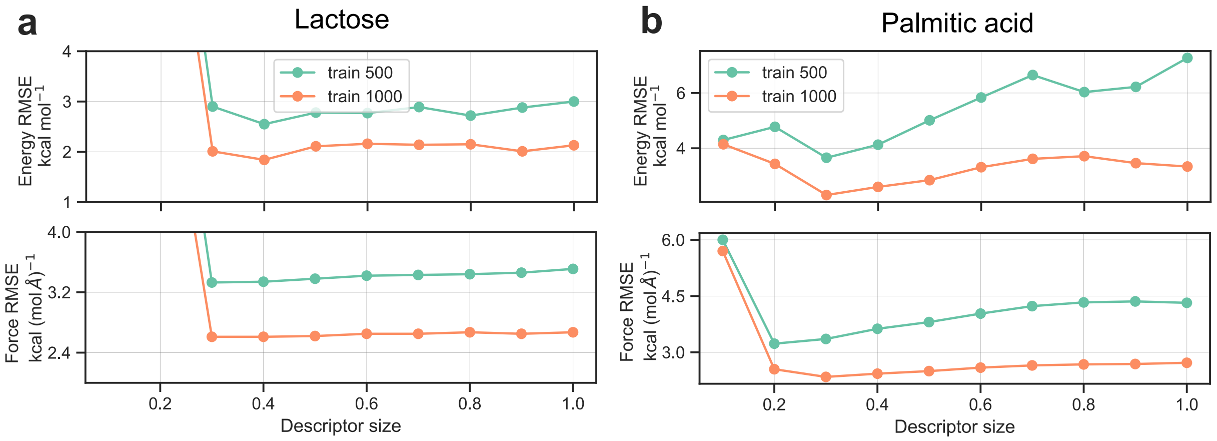

Supplementary Figure 2. Accuracy of the models during the course of reduction. Energy (in kcal mol^{-1}) and force (in kcal (mol Å)$^{-1}$) root means square errors (RMSE) as a function of the size of the descriptor for lactose (**a**) and palmitic acid (**b**) trained with 500 and 1000 training examples (green and orange colors, respectively). Descriptor sizes in x-axis go from 1 to 0, where 1 corresponds to a default global descriptor and 0 to an empty descriptor.

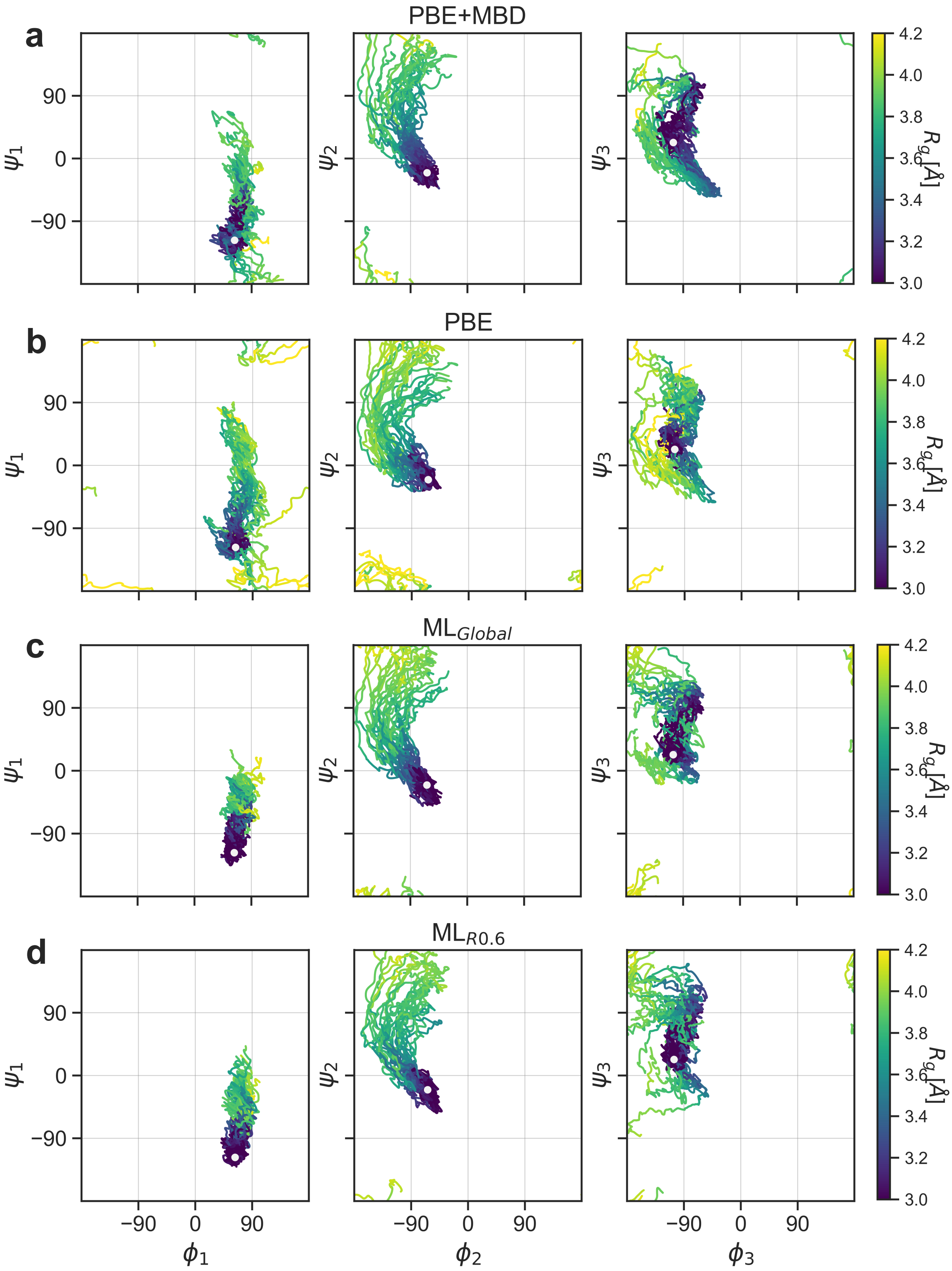

Supplementary Figure 3. Ramachandran plots for 30 external-force simulations for the Ac-Ala3-NHMe at 300K employing the PBE+MBD (**a**) and PBE (**b**) level of theory, as well as ML_{Global} (**c**) and $ML_{R0.6}$ (**d**) models trained on 5000 data points. Light grey dot represent starting configuration.

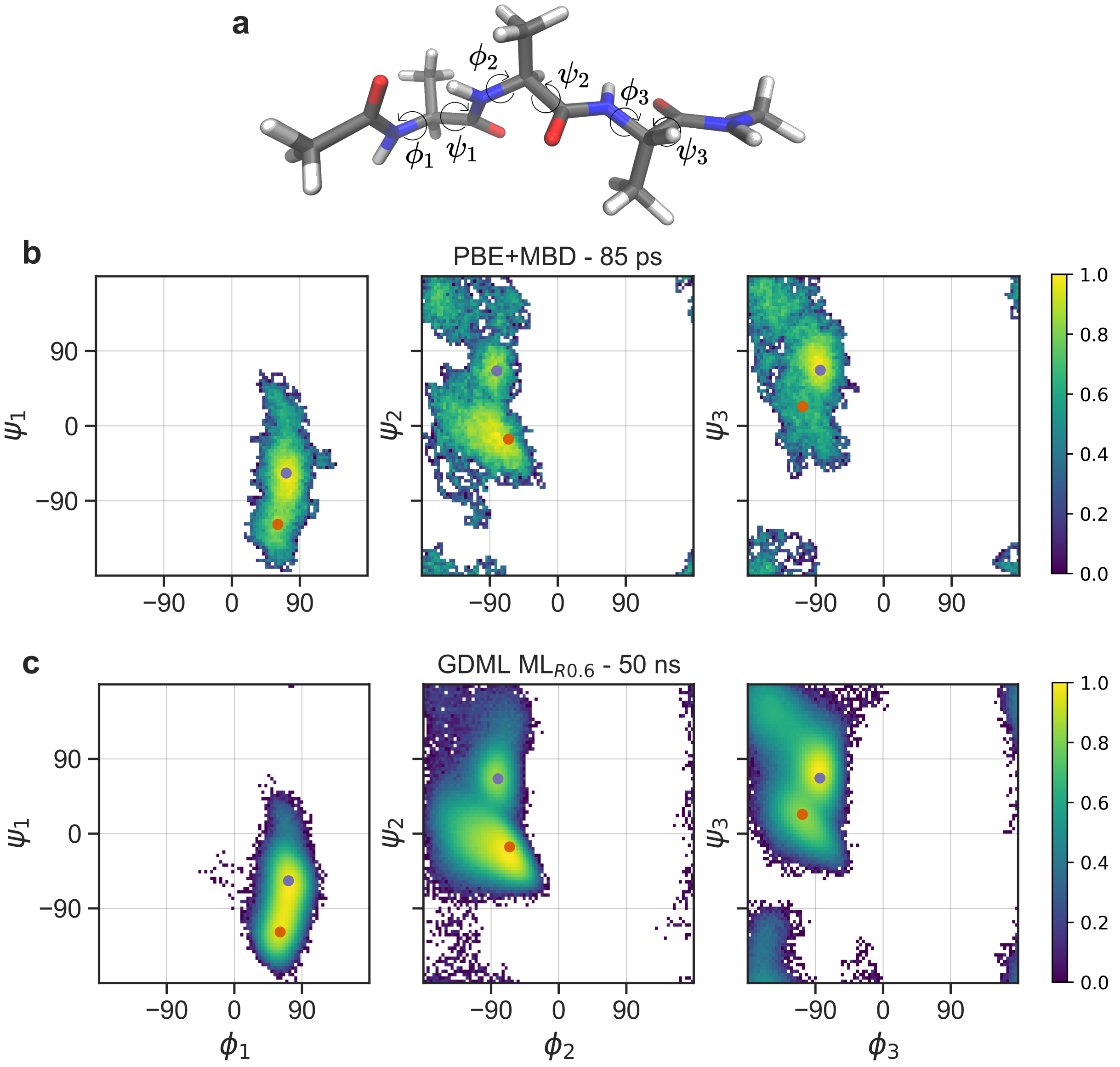

Supplementary Figure 4. Ramachandran plots with angles ϕ/ψ defined in (**a**). Ramachandran plots show the initial dataset of Ac-Ala3-NHMe - 85 ps, computed at the PBE+MBD level of theory (**b**), along with the resulting 50 ns dynamics obtained with the reduced $\mathrm{ML}_{R0.6}$ model trained on 5000 data points (**c**). The color represents the population of the bins on a log scale normalized to the 0-1 range. Orange and purple dots indicate compact and extended structures, respectively.

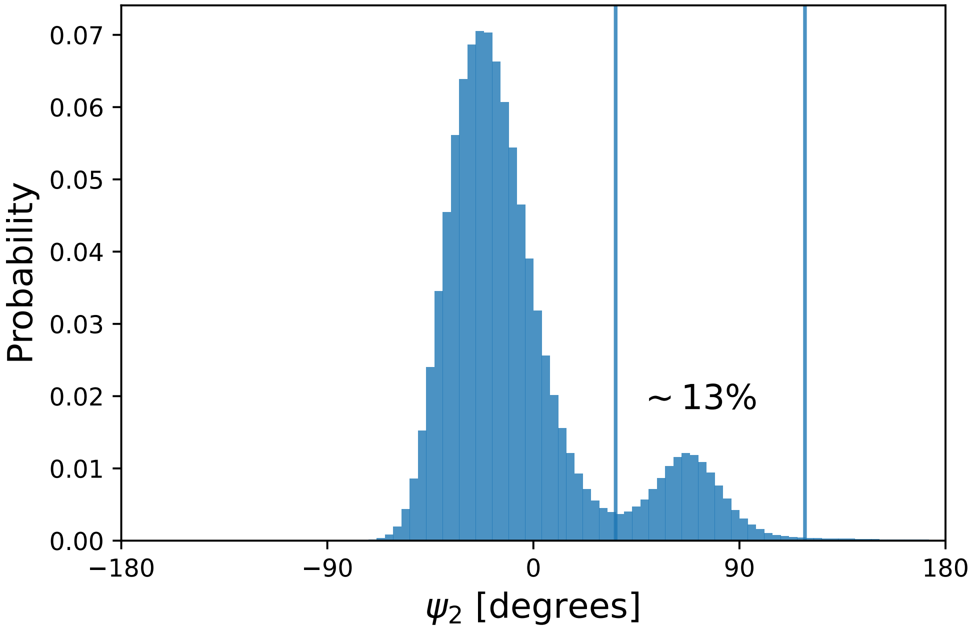

Supplementary Figure 5. Distribution of ψ_2 angle during 50 ns dynamics of Ac-Ala3-NHMe. Two vertical lines mark out region of configurations in extended state.

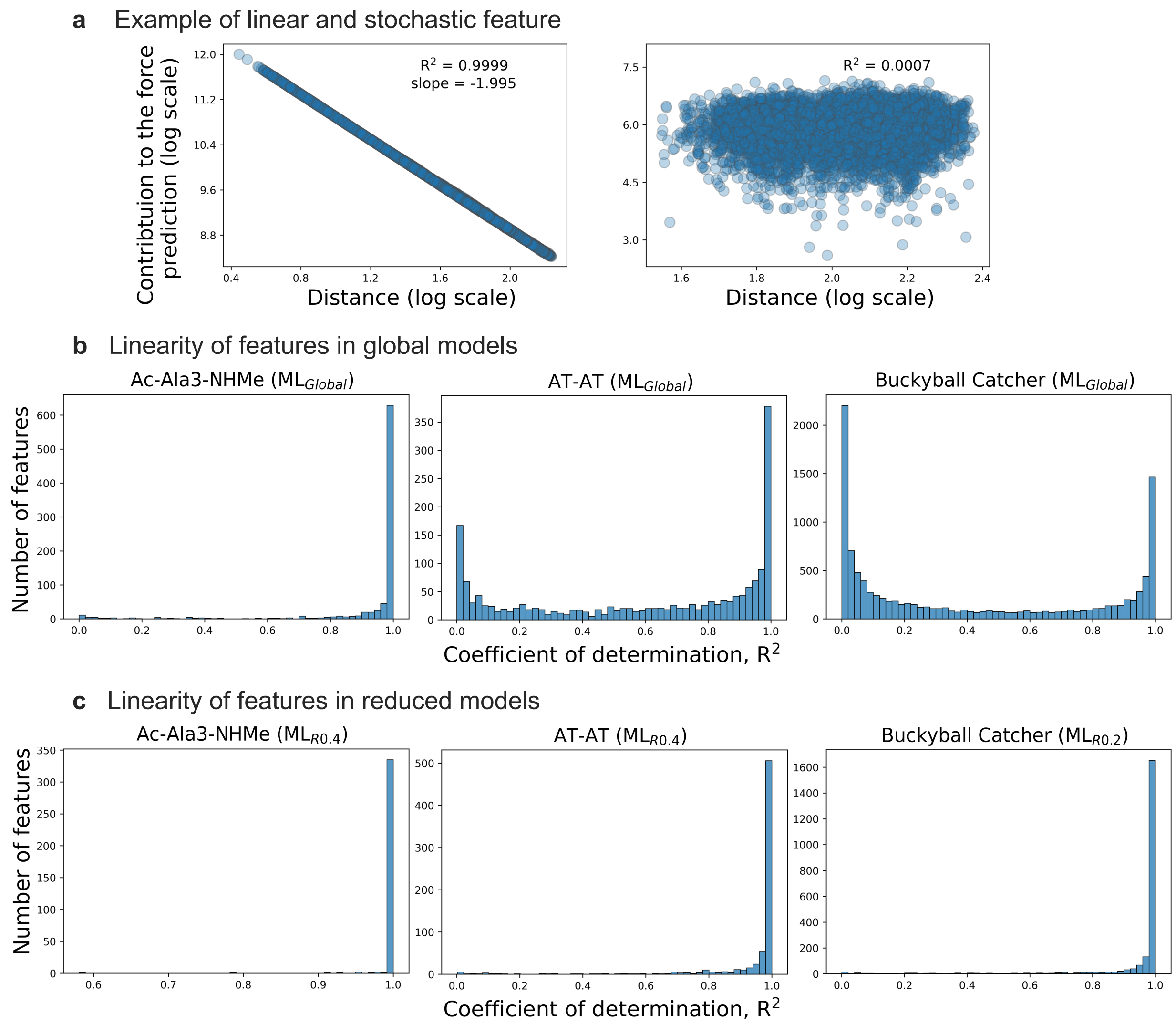

Supplementary Figure 6. Analysis of the scale of the contribution with respect to distance of particular features in the global and reduced models. Example of linear and stochastic features (**a**), linearity of features in global (**b**) and reduced (**c**) models.
